# Adipose-Derived Stromal Vascular Fraction Cell Effects on a Rodent Model of Thin Endometrium

**DOI:** 10.1371/journal.pone.0144823

**Published:** 2015-12-14

**Authors:** Robert K. Hunter, Chris D. Nevitt, Jeremy T. Gaskins, Bradley B. Keller, Henry C. L. Bohler, Amanda J. LeBlanc

**Affiliations:** 1 Department of Obstetrics, Gynecology and Women’s Health, Division of Reproductive Endocrinology and Infertility, University of Louisville School of Medicine, Louisville, Kentucky, United States of America; 2 Department of Biochemistry and Molecular Genetics, University of Louisville School of Medicine, Louisville, Kentucky, United States of America; 3 Department of Bioinformatics and Biostatistics, University of Louisville School of Public Health and Information Sciences, Louisville, Kentucky, United States of America; 4 Cardiovascular Innovation Institute, Louisville, Kentucky, United States of America; 5 Department of Physiology, University of Louisville School of Medicine, Louisville, Kentucky, United States of America; Queen's University, CANADA

## Abstract

Endometrial dysfunction affects approximately 1% of infertile women, and there is currently no standard therapy for improving fertility treatment outcomes in these patients. In our study, we utilized a rodent model of thin endometrium to test whether intrauterine application of adipose-derived stromal vascular fraction cells (SVF) could improve morphological and physiological markers of endometrial receptivity. Using anhydrous ethanol, endometrial area and gland density were significantly reduced in our model of thin endometrium. Application of SVF was associated with a 29% reduction in endometrial vascular endothelial growth factor (VEGF) expression and significant increases in uterine artery systolic/diastolic velocity ratios and resistance index values, suggesting reduced diastolic microvascular tone. However, no significant improvements in endometrial area or gland density were observed following SVF treatment. 3D confocal imaging demonstrated poor engraftment of SVF cells into recipient tissue, which likely contributed to the negative results of this study. We suspect modified treatment protocols utilizing adjuvant estrogen and/or tail vein cell delivery may improve SVF retention and therapeutic response in subsequent studies. SVF is an easily-obtainable cell product with regenerative capability that may have a future role in the treatment of infertile women with endometrial dysfunction.

## Introduction

Endometrial thickness is the primary clinical metric for assessing uterine receptivity during assisted reproduction cycles. Measured by in-cycle transvaginal ultrasound, a number of studies have shown that a bilayer thickness of less than 7 mm on the day of HCG administration is associated with suboptimal treatment outcomes [1–4], and the incidence of persistent thin endometrium has been estimated at approximately 1% of patients undergoing in vitro fertilization therapy [5–6]. A number of adjuvant treatments have been utilized to address this issue when it is encountered, including supplemental administration of estradiol [7,8], low-dose aspirin [9], vaginal sildenafil citrate [10,11], combination pentoxifylline and tocopherol [12], gonadotropin releasing hormone agonist [13], and human chorionic gonadotropin [14]. Others have attempted in-cycle endometrial biopsy [15–17], intrauterine granulocyte colony-stimulating factor (G-CSF) instillation [6,18], and pelvic floor neuromuscular electrical stimulation [19]. Unfortunately, none of these therapies has yet shown reliable improvements in pregnancy outcomes, and persistent thin endometrium continues to cause significant treatment challenges for both patients and providers alike.

Recently, increasing attention has been given to the inherent regenerative capacity of the endometrium. During a woman’s reproductive years, the endometrium may undergo more than 400 cycles of growth, differentiation, and shedding, and dynamic changes also occur following events such as parturition, endometrial resection, and hormone replacement therapy [20,21]. Adult stem cells maintain cellular production in tissues with this capacity for continuous regeneration [20], and populations of native progenitor cells with specific markers have recently been identified in both the functionalis and basalis layers of the endometrium [22–24]. It follows that a number of endometrial pathologies, including endometriosis, Asherman syndrome, intrauterine adhesions, and persistent thin endometrium may all either be caused or potentiated by dysfunction of native endometrial stem cells [25].

Relatively few studies have investigated therapies that might improve progenitor cell function at the level of the endometrium, but significant progress has been made in other fields, including cardiovascular medicine, plastic surgery, hematology, gastroenterology, neurology, urology, and orthopedics [26]. Adipose tissue contains a heterogeneous mesodermal cell population defined as the stromal vascular fraction (SVF), which has increasing clinical interest due to its ability to differentiate into various functional lineages [27]. The SVF is typically harvested via enzymatic digestion and centrifugation, and contain a heterogeneous array of cell types including endothelial cells, perivascular cells, fibroblasts, immune cells, and mesenchymal stem cells [28]. This mixed cell population can be systemically delivered, and has been shown to incorporate into peripheral vasculature and tissues with therapeutic effects throughout the body in a rodent model [29]. In poorly vascularized tissues, such as ischemic myocardium, local delivery of SVF has been shown to be a viable alternative for improving injured tissue recovery and function [30,31]. These effects are likely due to the proficient vasculogenic (new vessels forming from single cells) and angiogenic (new vessels forming from existing vessels) capabilities of freshly isolated and minimally cultured SVF following implantation in the periphery [32,33]. These vascular activities along with other favorable characteristics such as its relative ease of isolation (versus other mesenchymal stem cell sources) and the ability to suppress inflammation [34] make SVF a desirable candidate for clinical regenerative therapies and the choice population for cell-based therapy in the present study.

Two chemical models of thin endometrium have been developed in rodents that have facilitated early investigations into cell-based regenerative therapies. Cocuzza et al. developed a model of thin endometrium using an intrauterine instillation of trichloroacetic acid, although two-thirds of the animals either died or had insufficient tissue for analysis after short-term follow up [35]. Recently, another study utilized a similar model to test the therapeutic effects of injections of intrauterine and intraperitoneal mesenchymal stem cells along with adjuvant estrogen treatment. Their results showed decreased rates of fibrosis and increased expression of vascular endothelial growth factor (VEGF), proliferating cell nuclear antigen (PCNA), and Ki-67 (another marker of cellular proliferation)[36]. An alternative model developed by Jing et al. utilized an intrauterine instillation of anhydrous (95%) ethanol, which showed a 70% success rate of inducing thin endometrium when retained for 5 minutes in an initial study [37]. These authors have subsequently shown improvements in endometrial histology and inflammatory marker expression with both systemic [38] and intrauterine [39] administration of bone marrow mesenchymal stem cells given on the day of injury. In humans, two publications have documented clinical improvements in Asherman syndrome patients following cell-based therapy. Nagori et al. reported improvements in endometrial thickness following uterine curettage and intrauterine instillation of bone marrow-derived stem cells; the patient later conceived via in vitro fertilization with donor oocytes [40]. Singh et al. described a case series of six patients with refractory Asherman syndrome who underwent a transvaginal sub-endometrial implantation of bone marrow-derived stem cells. Five of the six patients resumed spontaneous cyclic menstruation by 3 months after the implantation [41]. To our knowledge, no previous studies have utilized adipose-derived SVF cells as a regenerative agent in gynecologic research.

The objective of the present study was to evaluate the therapeutic potential of adipose-derived SVF cells on a rodent model of persistent thin endometrium. We chose to utilize the anhydrous ethanol model of thin endometrium developed by Jing et al. [37] in our study due to its comparable pathophysiological features to thin endometrium in humans [42]. Previous work with this model has demonstrated the benefit of bone marrow-derived stem cells when given on the day of endometrial injury [38,39], and we sought to expand on these initial studies by increasing the time interval between injury and treatment to better reflect the chronic state of endometrial dysfunction in human infertility patients [43]. We chose to work with adipose-derived SVF cells rather than a bone marrow-derived cell product due to its relative ease of isolation and high therapeutic potential for use in humans [44]. We hypothesized that SVF applied to a rodent model of persistent thin endometrium would improve both morphological and physiological markers of endometrial receptivity.

## Methods

### Subjects

Four to six month-old female Sprague Dawley rats (Harlan Laboratories) weighing 250–300 grams were used in all experiments. The rats were housed in groups with free access to food and water and were maintained on a regular 12-hour light/dark cycles. After acclimating to facility conditions for a minimum of two weeks, daily morning vaginal cytology was used to assess estrous cycle frequency and regularity. Thirty rats with regular 4 to 5 day estrous cycles were selected for inclusion in our study, and all subsequent procedures were performed during the estrus phase of the cycle. All animal surgeries were performed in accordance with protocols approved by the University of Louisville Institutional Animal Care and Use Committee and the *Guide for the Care and Use of Laboratory Animals* (8^th^ ed., 2011). In all animal surgeries, adequate anesthesia was maintained by monitoring heart rate, respiratory effort, and tactile toe pinch reflex.

### SVF Cell Isolation and Culture

**Fig 1:**
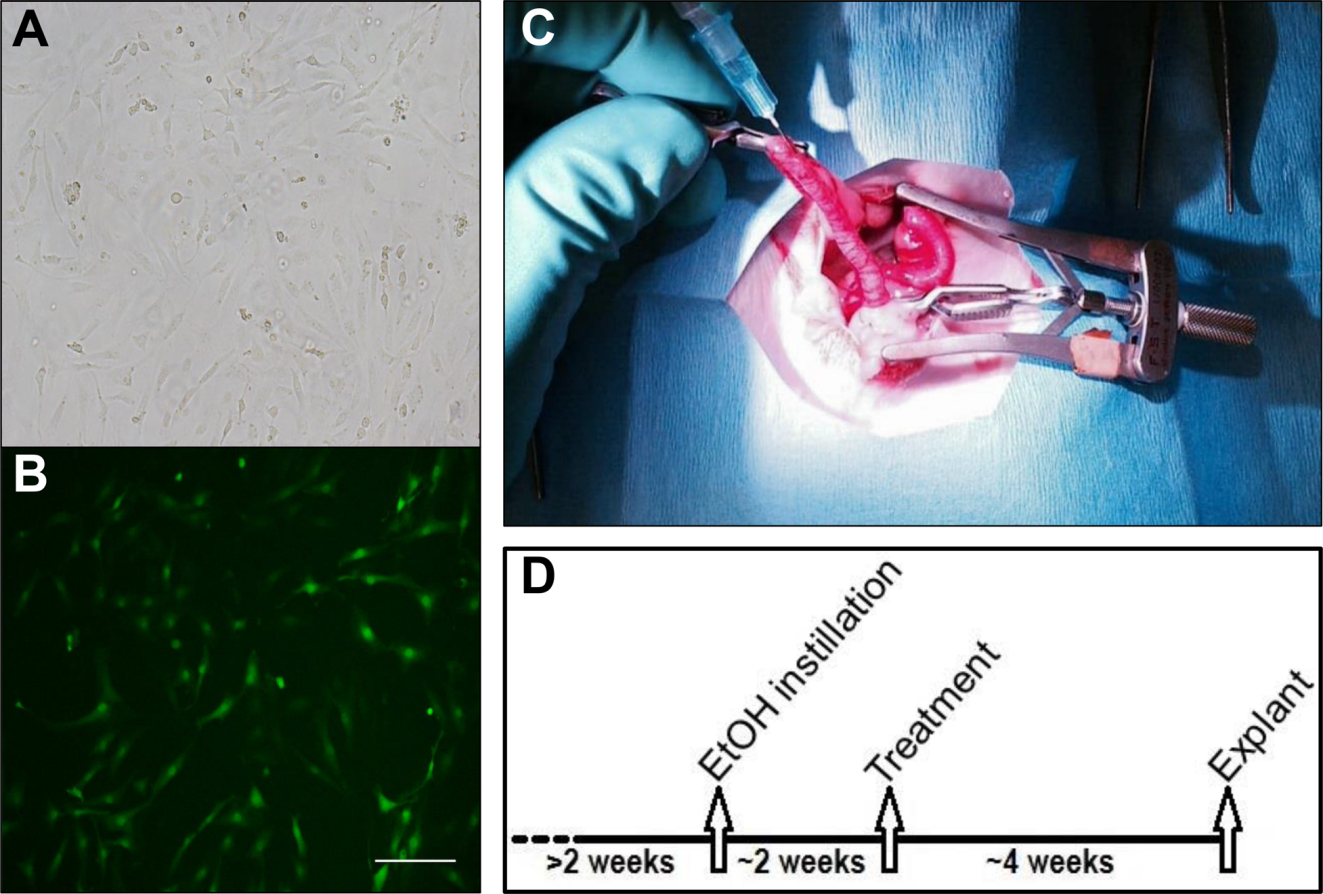
(A) Cultured stromal vascular fraction (SVF) cells viewed under bright field microscope at 10x magnification. (B) Fluorescent imaging demonstrates green fluorescent protein (GFP+) signal from SVF cells. Scale bar = 100 μm. (C) Intrauterine instillation procedures were performed with proximal and distal hemostatic clamps in place to isolate each uterine horn. (D) Protocol timeline demonstrates the approximate schedule of injury, treatment, and analysis. Exact dates were dependent on individual estrous cycles.

SVF was isolated and pooled from a population of 5 and 6 month-old green fluorescent protein (GFP+) transgenic female Sprague Dawley rats (n = 4) as described previously [30,31]. Briefly, parametrial fat pads were surgically excised and placed into phosphate-buffered saline with 0.1% bovine serum albumin (PBS-BSA). Major blood vessels were removed, and the remaining tissues were minced until grossly homogenous in appearance. The tissues were then incubated at 37°C and agitated with type I collagenase (3 mg/mL of tissue) and DNAase (1.5 mg/mL of tissue) for 40 minutes. The digest was then centrifuged at 350g for 4 minutes to allow separation of its component layers. The adipocytes and supernatant were removed, and the remaining pellet was resuspended in PBS-BSA. This suspension was centrifuged at 300g for 4 minutes, and the resulting supernatant was again removed. The remaining pellet containing the stromal vascular fraction was suspended in sterile PBS, and nucleated cell concentrations were measured using an automated cell counter (Nexcelom). Aliquots of 5x10^6^ cells in 1 mL of Recovery Cell Culture Freezing Medium (Life Technologies) were stored at -80°C until needed. For instillation procedures, cells were plated in 1% gelatin-coated T25 flasks at a density of 2.5x10^6^ cells/flask in Rat Complete Medium (Low Glucose DMEM supplemented with 10% FBS, L-Glutamine, ECGS, and HEPES) and grown to confluence (Fig 1A and 1B). Upon reaching confluence, cells were trypsinized, pelleted, resuspended in PBS, and counted by the previously described method. After determining cell counts, the cell suspension was divided, pelleted, and resuspended in PBS at a concentration of 1x10^6^ cells/mL. In order to allow distinction between effects that occurred due to SVF administration and those that may have resulted from an inflammatory response to a non-specific cell product [45], nonviable SVF (nvSVF) was generated by suspending cells in 70% methanol for 3 hours after counting, followed by pelleting and resuspending in the appropriate volume of PBS.

### Ethanol Instillation Procedure

Rats were anesthetized using a 3–5% isoflurane-O_2_ balance. Animals were able to breathe spontaneously during the procedure, and intubation was not required. Following the induction of anesthesia, rats were placed in supine position and the inferior abdomen was shaved and disinfected with 70% ethanol and 2% chlorhexidine diacetate (Nolvasan®) solutions. Ketoprofen (5 mg/kg) was injected subcutaneously to provide post-operative analgesia. After confirming adequate anesthesia by loss of toe-pinch reflexes, a transverse lower abdominal incision was made sharply and carried down to the peritoneal cavity. The uterine horns were identified and confirmed to have grossly normal anatomy. Hemostatic clamps were placed distally and proximally on the left horn, and approximately 0.5 mL of 90% ethanol was instilled into the lumen of the horn using a 25 gauge syringe (Fig 1C). Clamps were removed after 5 minutes, and the right horn was clamped in a similar fashion. After isolating the right horn, the left horn was flushed with sterile PBS to dilute and remove any residual ethanol, and the right horn was instilled with PBS for 5 minutes via syringe. Clamps were then removed from the right horn, and the peritoneum was irrigated with lactated Ringer’s solution. At the conclusion of the procedure, the abdominal wall was closed with 4–0 polyglactin 910 (Vicryl®) suture and surgical clips. 5 mL of lactated Ringer’s solution was injected subcutaneously to provide post-operative hydration. Additional ketoprofen (5 mg/kg) was provided for 48 hours to provide post-operative analgesia.

### Treatment Groups

Rats were allowed to recover from the ethanol instillation procedure for a duration of three estrous cycles (approximately two weeks). Fig 1D displays the timeline of experimental groups. During the third estrus phase following their initial surgery, all rats underwent a second surgical procedure. To confirm initial uterine injury at the time of the treatment, 10 of the rats were euthanized and underwent uterine explant to allow for histological analysis prior to further intervention. Nineteen additional rats underwent a second survival surgery similar to the one described above, where each uterine horn was instilled with varied treatments to create the following experimental groups:

- **Injured-Control:** injured horns were instilled with 0.5 mL of PBS-BSA, n = 8
- **Sham-Control:** uninjured horns were instilled with 0.5 mL of PBS-BSA, n = 11
- **Injured-SVF:** injured horns were instilled with a suspension of SVF cells (1x10^6^ cells/0.5mL), n = 8
- **Sham-SVF:** uninjured horns were instilled with a suspension of SVF cells (1x10^6^ cells/0.5mL), n = 8
- **Injured-nvSVF:** injured horns were instilled with a suspension of nonviable SVF cells (1x10^6^ cells/0.5mL), n = 3

As before, all instillations were limited to a specific uterine horn with hemostatic clamps and were retained for 5 minutes. Post-operative care was similar to the previous surgery.

### Uterine Explant Surgery

All rats in the experimental groups were followed for four weeks post-operatively and then sacrificed during a subsequent estrus phase. All sacrificed rats underwent uterine explant by laparotomy immediately after cervical dislocation. The left and right uterine horns were identified and sharply dissected from all surrounding adnexal structures. Each horn was divided into two halves in its mid-isthmic region, and resulting specimens were placed into either 10% formalin or 4% paraformaldehyde (PFA) for subsequent analyses.

### Histological and Immunohistochemical Analyses

To assess for differences in gross morphologic features, histologic analysis was performed. Uterine horns were removed from buffered formalin solution, dehydrated in graded alcohol solutions, and embedded in paraffin. Transverse sections of the uterus were sliced at a thickness of 6 μm, and hematoxylin-eosin staining was applied to observe gross tissue structures. Endometrial area and the percentage of the uterine wall comprised by the endometrium were calculated from representative slides using ImageJ software (NIH, version 1.48). Endometrial gland counts and gland densities (gland count / endometrial area) were also assessed from representative slides.

For immunohistochemistry, slides of uterine tissue were deparaffinized in xylene and rehydrated by a series of washes with descending ethanol concentration. Tissues were permeabilized with 0.5% Triton-X-100 in PBS followed by antigen retrieval in Dako solution for 20 minutes at 100°C. Blocking was performed with 10% goat serum before overnight incubation with rabbit anti-VEGF antibody (Abcam, ab46154) at a 1:100 dilution. Negative control tissues received rabbit IgG at 1:100 in place of primary antibody. Goat anti-rabbit 488 secondary antibody (Santa Cruz) was applied for 1 hour at a concentration of 1:200 followed by nuclear staining with DAPI at 1:10,000. Anti-fade solution was applied before coverslips were placed on the tissue slides. Four images were taken per slide at 10x magnification with 350 nm, 490 nm, and 557 nm excitation filters. ImageJ (NIH, version 1.48) was used for the subsequent image analysis. VEGF, dextran-rhodamine, and DAPI signal intensity were measured for each image. Signal intensity values were taken from histograms corresponding to the appropriate channel for each object of interest in the entire field of view for each image. Intensities from each image were averaged to obtain representative sample values. Negative controls were used to subtract out background signal for VEGF.

### Protein Isolation and Western Blot

Formalin-fixed uterine tissue underwent protein isolation using the Qproteome FFPE Tissue Kit (Qiagen) according to manufacturer specifications. Sample recovery was performed by mincing tissue in lysis buffer containing 1mM DTT, 0.1% Protease Inhibitor (Sigma, P-8340), and 0.81 mM NaOrthovanadate. The resulting mixture was centrifuged and the supernatant was saved. Protein concentrations were determined through a Bradford Protein Quantification assay (BioRad). Samples were brought to a concentration of 1 μg/μL with lysis buffer. 15 μL of each sample was mixed 1:1 with 2x Laemmli buffer containing 5% β-mercaptoethanol and were heated for 5 minutes at 95°C. Pre-cast 4–20% gradient gels (BioRad) were loaded with 30 μL of each sample. 10 μg of rhVEGF (Abcam, ab55566) was loaded as a positive control. Gels were run at 200V for 40 minutes. Transfer to a PVDF membrane was then carried out overnight in a cold room at 25V. Membranes were blocked for 1 hour in 5% milk before incubating with primary antibodies overnight. Mouse anti-β-actin (Santa Cruz) was used at a 1:2000 dilution and Rabbit anti-VEGF (Abcam, ab46154) at 1:1000 dilution. The fluorescent secondary antibodies Goat anti-mouse 488 and goat anti-rabbit 594 were prepared at a 1:1,000 dilution and applied for 1 hour. Imaging was performed on a GE Typhoon with the following settings: VEGF– 532 nm laser, 610 nm bandpass, 400V PMT; β-actin– 488 nm laser, 520 nm bandpass, 300V PMT. Densitometry was performed using ImageJ software (NIH, version 1.48), with VEGF being normalized to β-actin as a loading control for each sample.

### Ultrasonographic Assessment

High-resolution transabdominal ultrasonography (Vevo2100, VisualSonics, Toronto, CA) was performed on 10 rats on the day of their ethanol instillation, SVF instillation, and uterine explant procedures. Rats were anesthetized using 5% isoflurane via nose cone and maintained on 2% isoflurane during the procedure. Heart rate and rectal temperature were continuously monitored. A 30 MHz probe was used to measure the antero-posterior diameter of each uterine horn at three points in the mid-isthmic region. Color flow Doppler was then used to locate the proximal uterine artery for each horn approximately 1cm from its junction with the uterine body. For pulsed wave Doppler velocity acquisition, the high pass filter was set at 20 Hz and the pulsed repetition frequency was set between 4 and 48 kHz to detect low to high blood flow velocities. Peak systolic velocity (PSV) and end-diastolic velocity (EDV) were measured from three consecutive cycles. Systolic to diastolic (S/D) ratio (PSV/EDV) and resistance index ([PSV–EDV]/PSV) values were then calculated for each uterine horn.

### 3-D Confocal Imaging

Fifteen minutes prior to explant, animals were given a tail vein injection of 3.0 mg dextran-rhodamine suspended in 1.5mL PBS to label vascular elements. Explanted uterine tissue was stored overnight in 4% PFA and transferred to 0.4% PFA the following day. For analysis, uterine horns were sectioned longitudinally to expose the luminal surface, permeabilized with 0.5% Triton-X-100, and nuclei were stained with RedDot2 (Biotium, #40061) at a 1:200 dilution. Tissue was placed in Fluoromount-G (Southern Biotech) and imaged on an Olympus BX61WI confocal microscope with a 20x objective (NA 0.5) using a 3 μm step size and total imaging depth of 120 μm. The following excitation/emission filter sets were utilized: GFP^+^ SVF signal–eGFP, 13% offset; dextran-rhodamine perfused vasculature–rhodamine, 11% offset; RedDot2 nuclear stain–AF647, 12% offset. 3-D reconstructions were generated from confocal images using AMIRA v6.0 software.

### Statistical Analysis

Endometrial area, the percent of uterine wall composed of endometrial tissue, VEGF expression, dextran-rhodamine expression, uterine horn diameter, change is S/D ratio, and change in resistance index are all analyzed using analysis of variance (ANOVA) with random effects to account for potential correlations in the horn of the same rat. Due to their non-normality, gland count and gland density are analyzed using non-parametric tests: median estimates, Kruskal-Wallis test for overall group differences, Wilcoxon test for paired comparisons, and bootstrap estimates for confidence intervals and standard error of the median. VEGF and dextran-rhodamine expressions are transformed to the log scale. All calculations were performed using the statistical software R [46].

## Results

### Morphologic Outcomes

**Fig 2:**
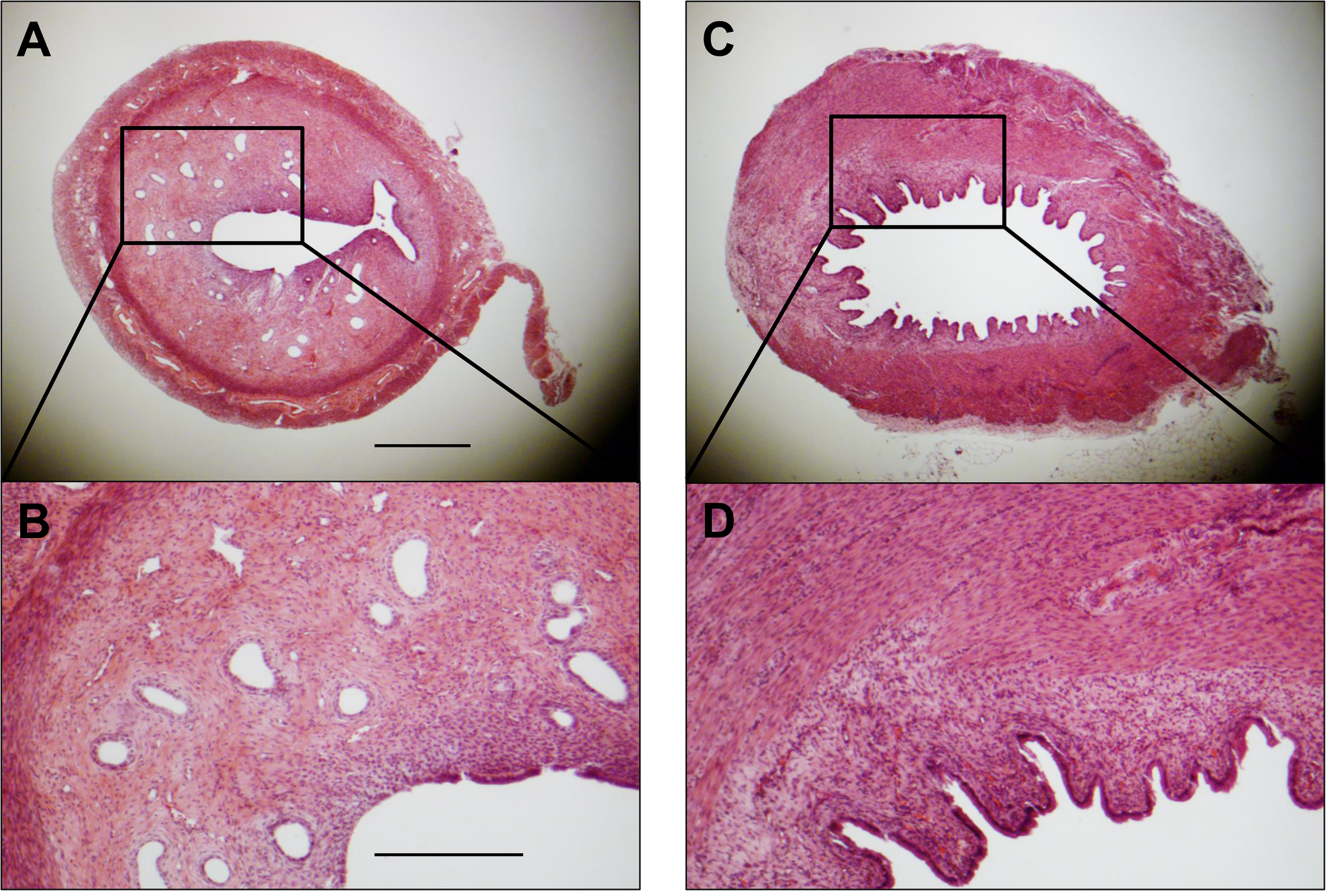
Representative examples of uterine horn cross sections in the third estrus phase following intrauterine instillation of either phosphate-buffered saline (PBS) (A and B) or ethanol (C and D). Gross differences in endometrial area and gland density are apparent. Scale bars = 500 μm (A) and 250 μm (B).

Ten rats were euthanized during the third estrus phase after undergoing the ethanol instillation procedure. This allowed us to characterized uterine features at the time of experimental intervention in the other groups (Fig 2). Injured horns showed trends towards reduced endometrial area and gland density, consistent with previous studies on endometrial injury [35,37], though statistical significance was not reached due to the small sample size. There were no unexpected complications or mortalities in this group.

**Fig 3:**
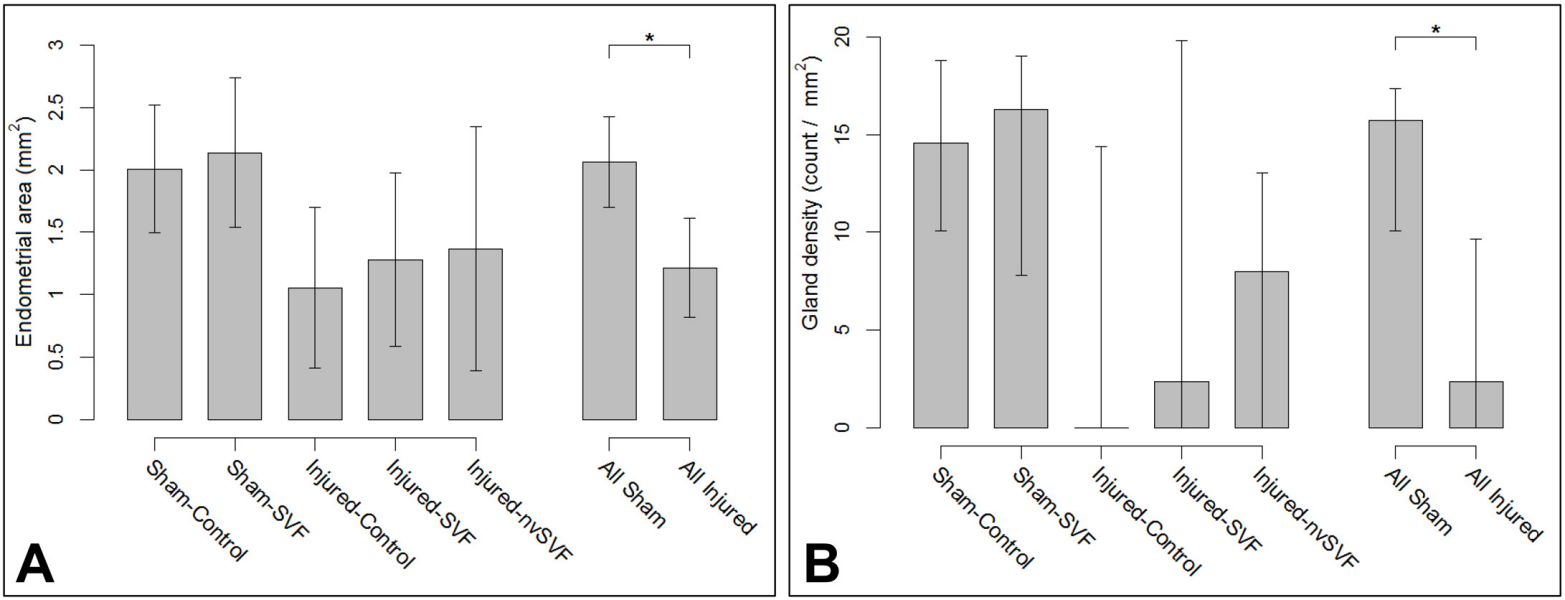
Graphs of mean endometrial areas (A) and median gland densities (B) between individual experimental groups (left) and collective sham and injured groups (right). Significant differences were observed between collective sham and injured groups (asterisks). Error bars represent standard deviation (A) and Q1, Q3 (B).

Following the experimental treatment surgery, significant histopathological differences were noted across groups following uterine explant. Mean endometrial area measurements (± standard error) were as follows: Sham-Control, 2.01±0.24 mm^2^; Sham-SVF, 2.14±0.29 mm^2^; Injured-Control, 1.05±0.31 mm^2^; Injured-SVF, 1.28±0.33 mm^2^; and Injured-nvSVF, 1.37±0.47 mm^2^. Significant differences across groups were found (p = 0.0335), but differences attributable to SVF therapy were not observed. Collectively, injured groups were found to have smaller endometrial areas than the sham groups by an average of 0.91 mm^2^ (p = 0.0043), as seen in Fig 3A. The percentage of the uterine wall comprised of endometrial tissue differed significantly across groups (p = 0.0006), with injured horns being an average of 24.8% lower than sham horns (p = 0.0002). No differences attributable to SVF treatment were noted.

Injured horns collectively contained fewer glands than sham horns (p = 0.0051), but no differences were independently observed based on SVF treatment (Sham-Control, 27±4.6; Sham-SVF, 30±9.1; Injured-Control, 0±8.9; Injured-SVF, 4.5±13.0; and Injured-nvSVF, 10±11.8). Adjustment of gland count for endometrial area showed that gland density did vary significantly across groups (p = 0.0451), with median and standard errors as follows: Sham-Control, 14.6±2.2; Sham-SVF, 16.3±3.4; Injured-Control, 0.0±4.9; Injured-SVF, 2.4±6.7; and Injured-nvSVF, 8.0±4.8. Differences in gland density between injured and sham horns was significant (p = 0.0026), although no effect of SVF treatment was seen.

### VEGF Expression

**Fig 4:**
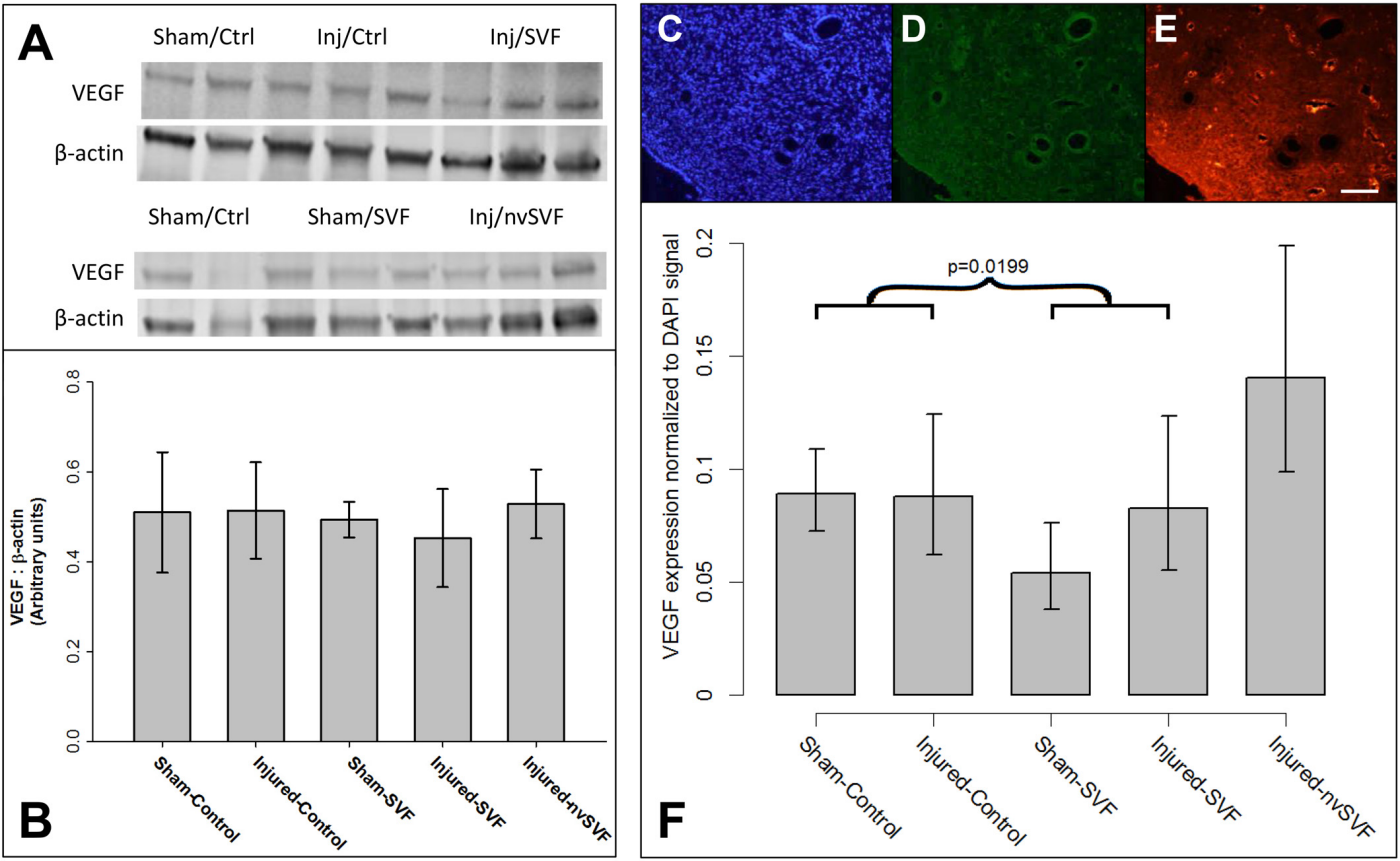
(A,B) Expression of vascular endothelial growth factor (VEGF) and β-actin measured by Western blot revealed no significant differences across experimental groups. Representative images of endometrial DAPI (C), green fluorescent protein (D), and rhodamine (E) expression measured by immunohistochemistry in an injured-control horn. A significant reduction in VEGF expression normalized to DAPI was collectively noted across experimental groups that received SVF treatment (asterisk), though no significant differences were noted between individual groups (F). Error bars represent standard deviation. Scale bar = 100 μm.

To evaluate potential changes in VEGF expression across experimental groups, VEGF protein was quantified by Western blot and not found to differ when normalized to total tissue protein levels approximated by β-actin measurements (Fig 4A and 4B). However, VEGF was also assessed by immunohistochemistry (Fig 4C–4E), and normalization to tissue DAPI signals showed a significant variation across experimental groups (p = 0.0029), as seen in Fig 4F. VEGF levels were estimated to be 29% lower in horns exposed to SVF compared to control (p = 0.0199). There was no reduction in VEGF expression based on ethanol exposure or nvSVF treatment, suggesting that this effect was a unique outcome of SVF treatment. Across all groups, VEGF signals were noted to be strongest in association with epithelial cells of the endometrial glands and lumen. Quantification of endometrial dextran-rhodamine signals showed no significant differences in tissue vascularization either as a function of ethanol exposure or SVF treatment.

### Ultrasound Studies

**Fig 5:**
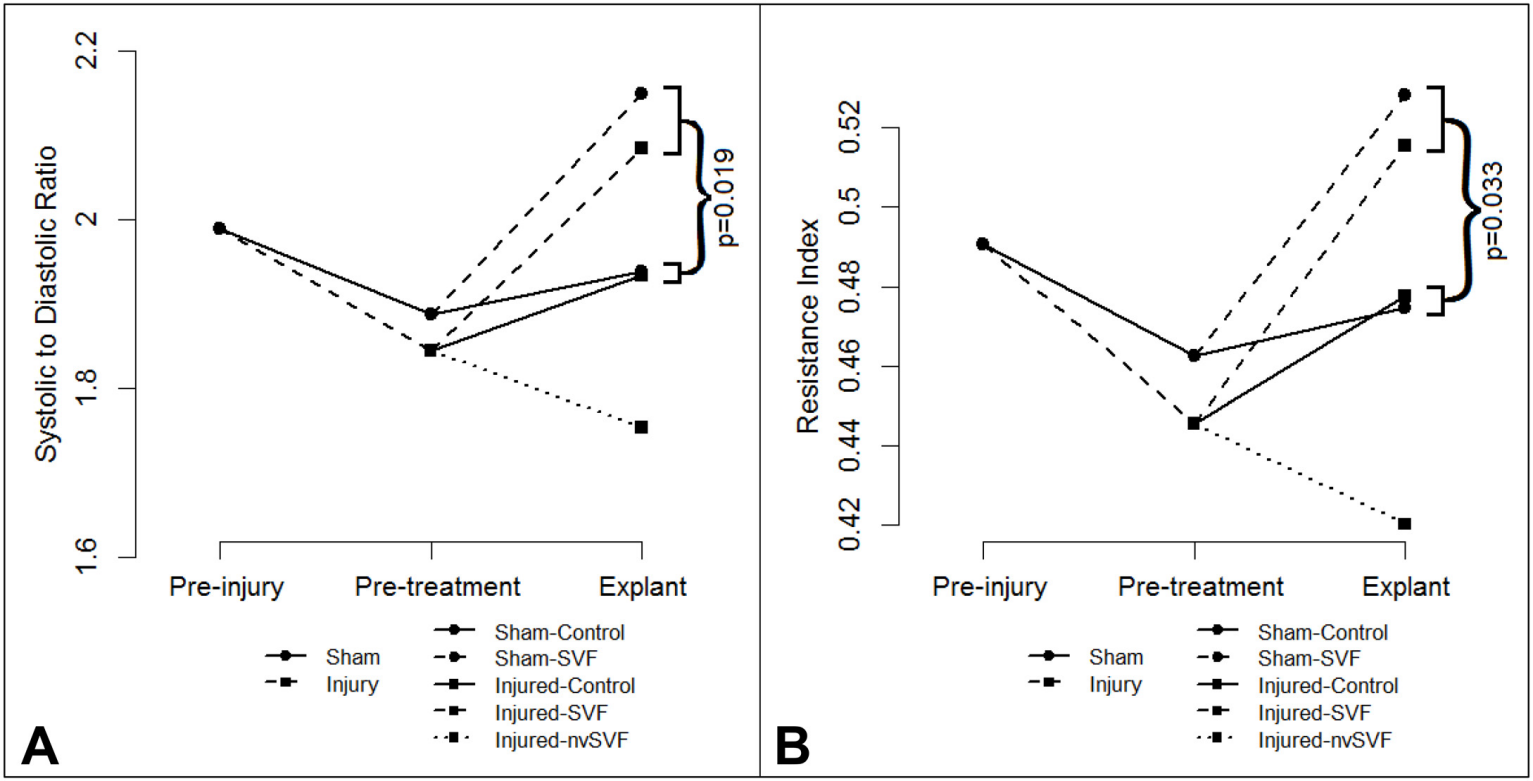
Mean systolic to diastolic (S/D) ratio (A) and resistance index (B) values plotted prior to injury, prior to treatment, and at explant. Increases in both metrics were noted in experimental groups that received SVF treatment, with changes in S/D ratio reaching statistical significance (p<0.05).

To assess whether morphometric or vascular changes could be detected by non-invasive imaging, ultrasound imaging was performed before ethanol instillation, immediately prior to treatment, and at the time of explant. Ultrasound analysis showed no significant differences in antero-posterior uterine horn diameters between the experimental groups at any of the time points studied. Doppler studies showed no significant changes in flow velocity waveform patterns following ethanol instillation compared to baseline values. However, following experimental treatment, S/D ratio values differed between the groups (p = 0.0296), with average (95% CI) interval changes as follows: Sham-Control, 0.018 (-0.29, 0.12); Sham-SVF, 0.323 (-0.12, 0.76); Injured-Control, 0.049 (-0.39, 0.49); Injured-SVF, 0.237 (-0.12, 0.60); and Injured-nvSVF, -0.048 (-0.41, 0.31). S/D ratios increased by an average of 0.252 in groups that received SVF (p = 0.0193), as shown in Fig 5A. Interval changes in resistance index values mirrored changes in S/D ratios (Fig 5B), with an average increase of 0.082 in groups that received SVF compared to control (p = 0.0328).

### SVF Cell Engraftment

To detect the presence of engrafted GFP+ SVF cells in recipient tissue, 3-D confocal imaging was performed. Confocal imaging demonstrated poor engraftment of SVF cells in treated specimens, as no nucleated GFP+ cells were identified after adjustment for background tissue auto-fluorescence.

## Discussion

Our study is the first to investigate the utility of adipose-derived SVF cells as a therapeutic agent in a rodent model of thin endometrium. Previous studies, including ours, have demonstrated that the SVF population used in the present study contains a large proportion of endothelial cells (~25%) [47–49], which exhibit the ability to promote vasculogenesis, angiogenesis, and vessel relaxation upon implantation [29,32,50]. Given its relative ease of isolation and established benefits in other areas of regenerative medicine [26], SVF has high potential for translational applications in human patients, possibly including women with endometrial dysfunction and subfertility. This initial study, however, failed to demonstrate clinically meaningful improvements in either morphological or physiological markers of endometrial receptivity in our rodent model of thin endometrium.

Histological analyses confirmed that intrauterine anhydrous ethanol instillation was effective in selectively damaging rodent endometrium, consistent with previous research [37]. By allowing a two-week recovery period between injury and treatment procedures, our model limited any potential confounding effects of acute inflammation on response to subsequent intervention. We feel that our chronological dissociation of injury and treatment allowed for a more appropriate model of persistent thin endometrium seen in human infertility patients, which is not associated with markers of acute inflammation and may rather lead to impaired fertility by altering endometrial microvasculature and perfusion [43].

Experimental instillation of SVF into injured uterine horns failed to demonstrate increases in endometrial area or gland density compared to our Injured-Control group. This observation contrasts with a recent report by Zhao et al. [39], which successfully demonstrated histological improvements from intrauterine instillation of bone marrow-derived mesenchymal stem cells given on the day of uterine injury modeling. Furthermore, in women with thin endometrium undergoing IVF treatment, intrauterine instillation of granulocyte colony-stimulating factor has been shown to improve markers of endometrial morphology, but not subsequent pregnancy rates [6]. These previous studies demonstrate that intrauterine administration of progenitor products may have a future role for improving endometrial morphology, despite the findings of our current study.

Our results also failed to demonstrate meaningful improvements in physiological markers of endometrial receptivity. We chose to focus on VEGF as our primary tissue marker as it has been shown to be an important regulator of endometrium-embryo crosstalk and neoangiogenesis around the time of implantation [51,52]; it may have reduced expression in women with endometrial dysfunction and otherwise unexplained infertility [53]; and its expression is reduced by anhydrous ethanol injury modeling in rodents [37]. We observed a counterintuitive reduction in endometrial VEGF expression following intrauterine SVF instillation. Endometrial perfusion, assessed by intravascular rhodamine signal intensity, did not differ between experimental groups. These findings contradict recent work by Kilic et al. [34], who demonstrated upregulation of VEGF expression and vascular proliferation in response to cell therapy administration. Of note, a trichloroacetic acid injury model was used in their study, and the investigators administered isolated adipose-derived mesenchymal stem cells into both the uterine horn and peritoneal cavity, along with adjuvant estradiol support. Despite these notable methodological differences, the authors successfully demonstrated the potential for functional marker improvement with a cell-based regenerative intervention.

The reasons for the discrepancies between our findings and those of other published studies may be elucidated by our ultrasound and confocal imaging data. SVF has been shown to reduce end-organ vasomotor tone when administered both locally [30] and systemically [29]. Our observation of altered uterine artery flow velocity waveform patterns following SVF administration, with widening between systolic and diastolic velocities, suggests reduced endometrial microvascular tone. We believe this reduction in resting microvascular tone may have increased tissue oxygen tension levels, which would be expected to reduce endometrial tissue VEGF expression [54], as we observed. Despite the changes in vascular dynamics noted after SVF administration, our observations with confocal microscopy suggest that no detectable amount of SVF cells remained successfully engrafted in recipient endometrium at the time of uterine explant. Due to the relatively high frequency of endometrial cell turnover with rodent estrous cycles, we suspect that modification of our protocol with either systemic cell delivery via the tail vein [38] or intrauterine cell delivery with adjuvant estradiol supplementation [36] may have resulted in higher rates of SVF cell retention than we were able to observe with the present study. SVF cells given via intrauterine instillation to spontaneously cycling recipients were likely rapidly cleared, which could have understandably contributed to the lack of observed responses seen in our study. However, many preclinical and clinical trials using stem cell delivery have noted persistent physiologic effects long after the implanted cells have disappeared [31].

There are several important limitations to consider when interpreting the results of our study. Most notably, all of the morphological and physiological markers of endometrial function used in our study are at best only surrogate markers of receptivity. Future studies using SVF to improve receptivity in rodents would benefit by observing mating and pregnancy outcomes in subjects that had been exposed to various combinations of injury and treatment, as these additional data points will provide a more direct assessment of endometrial function. Also, it should be noted that the physiological effects of SVF treatment in our study were likely limited by the poor rate of engraftment into host endometrium. We believe that future studies should either use a tail vein approach to SVF delivery [38], use estradiol to suspend the estrous cycle in a state of continued proliferation [36], or perhaps a combination of both. With improved SVF retention and engraftment, we suspect a more pronounced profile of treatment effects will be observable, leading to further insights that may highlight a path to potential translational applications in human patients.

## Conclusions

Our study successfully demonstrated an effective model of persistent thin endometrium in rodents using anhydrous ethanol. Experimental treatment with intrauterine SVF instillation did not produce meaningful improvements in morphometric or functional markers of endometrial receptivity. High resolution ultrasound efficiently provided vascular data, and SVF treatment did appear to reduce diastolic microvascular tone, increased tissue flow, and is consistent with the observed reduction in tissue VEGF expression. Tissue effects of SVF were likely limited due to poor cellular retention and engraftment. We believe future investigations into the use of SVF as a treatment for thin endometrium should include data collection of mating and pregnancy outcomes and further suggest that intravenous cell delivery and adjuvant estrogen administration be considered as potential means for improving cell retention and therapeutic potential.

## Supporting Information

S1 Dataset Raw data files generated during the study period. (ZIP)
